# Psychometric evaluation of the Persian version of the Hospital Survey on Patient Safety Culture Questionnaire (HSOPSC) in neonatal intensive care units: a methodological study

**DOI:** 10.1186/s12912-023-01435-1

**Published:** 2023-08-23

**Authors:** Mohadese Babaie, Manijeh Nourian, Foroozan Atashzadeh-Shoorideh, Houman Manoochehri, Malihe Nasiri

**Affiliations:** 1 Student Research Committee, Department of Pediatric and Neonatal Intensive Care Nursing, School of Nursing and Midwifery, Shahid Beheshti University of Medical Sciences, Tehran, Iran; 2 Department of Anesthesiology, School of Allied Medical Sciences, Alborz University of Medical Sciences, Karaj, Iran; 3 Department of Pediatric and Neonatal Intensive Care Nursing, School of Nursing and Midwifery, Shahid Beheshti University of Medical Sciences, Tehran, Iran; 4 Department of Psychiatric Nursing and Management, School of Nursing and Midwifery, Shahid Beheshti University of Medical Sciences, Tehran, Iran; 5 Department of Basic Sciences, School of Nursing and Midwifery, Shahid Beheshti University of Medical Sciences, Tehran, Iran; 6 Department of Biostatistics, School of Nursing and Midwifery, Shahid Beheshti University of Medical Sciences, Tehran, Iran

**Keywords:** Factor analysis, Neonatal intensive care units, “Hospital Survey on Patient Safety Culture” scale

## Abstract

**Background:**

A valid and reliable tool compatible with the culture is needed to evaluate the safety culture as one of the vital and promotional components in improving the quality of safety and health care. This study aimed to investigate the psychometric properties of the Persian version of the “Hospital Survey on Patient Safety Culture (HSOPSC)” in physicians and nurses working in Neonatal Intensive Care Units.

**Methods:**

In this methodological research, the qualitative face, content validity, and construct validity were performed by Confirmatory Factor Analysis to the psychometric evaluation of the HSOPSC questionnaire. Based on convenience sampling and the inclusion criteria, 360 individuals completed the questionnaire. Internal consistency and stability were measured. Data analysis was performed using SPSS 21 and LISREL.

**Results:**

In examining the construct validity, fit indices were not appropriate for the 12-dimension model of the Persian version. According to T-value, six heterogeneous items and a dimension were omitted. The 11-dimension model with 36 items showed an appropriate fit with the data. Cronbach’s alpha was evaluated at 0.79, and the stability was 0.82 (p˂0.001).

**Conclusion:**

The Persian version of HSOPSC with 11 dimensions and 36 items has favorable validity and reliability and can be used in NICUs.

## Introduction

Due to the sensitive and complicated nature of care in NICUs, neonatal safety is the main concern of the health system, and its goal is to reduce the incidence of errors, patient harm, and mortality [1]. These wards are a potentially error-prone environment, with 78% of infants experiencing at least one or more errors during hospitalization and suffering the consequences [2]. The prevalence of error and patient harm in NICUs has been increasing in recent years; even minor errors can lead to short-term and long-term devastating consequences, prolonged hospital stays, temporary disorders, or disability [3]. In Iran, despite the great emphasis on providing safe and quality care [4], the rate of errors and traumatic events harming patients is still reported to be high [5]. While 56% of all adverse events that occur are preventable. Therefore, maintaining patient safety is of particular importance [6].

Safety culture is one of the most influential factors in infant safety and reduces the fruitful and underlying factors leading to errors. As one of the vital components of healthcare safety promotion and improvement organizations [7], safety culture minimizes adverse events, possible harm due to improper care, length of hospital stay, readmissions, and mortality [8]. The safety culture establishment in NICUs influences clinical approaches in a way that justifies the differences in clinical care and its consequences [7], and the lack of it is the main obstacle to promoting safety in patient care.

## Background

Safety culture is a multidimensional concept that is completely dependent on the staff’s culture, beliefs, values, and attitudes [9]. Positive safety culture in the healthcare system is an assurance for the execution of treatment protocols with an emphasis on maintaining neonates’ safety [7]. As NICUs provide care to infants with the help of advanced devices and in a complex environment, there is a higher possibility of jeopardizing infant safety. Therefore, experts emphasize the accurate measurement of safety culture as a factor that can improve neonatal safety. In fact, measuring safety culture with a valid and reliable tool in the NICU, with its own conditions and sensitivity, can be useful in preventing errors and improving the level of safe care.

One of the various tools designed to measure safety culture is the 42-item *Hospital Survey on Patient Safety Culture (HSOPSC)* questionnaire developed in the US society by the Agency for Healthcare and Quality (AHRQ) in 2004 [10]. In this 12-dimensional tool, two dimensions are outcome measures. Seven dimensions examine the level of units, and others target the hospital level. The pilot survey of this tool, examining 1437 hospital workers, reported the tool’s acceptable levels of internal reliability (Cronbach’s α = 0.63–0.84) and construct validity [11].

Although this questionnaire has an American origin, it has been recognized and used as a suitable tool in diverse societies. The psychometric characteristics of HSOPSC had investigated in many studies in the past few decades [12–19]. In some societies, the structure of 12 dimensions is approved. In other, a construction with entirely different factors has been obtained.

Generally, various studies demonstrate that HSOPSC is the most widely used tool in the square of patient safety. Moreover, HSOPSC has been translated into 43 languages in the world, according to the latest report by 2022. In other words, about one hundred countries with diverse nationalities and cultures from the world’s continents have used HSOPSC to evaluate the safety condition in healthcare systems [20].

This tool is the most accurate and common tool for studying hospital safety culture [12, 16], which evaluates the safety culture at the level of hospitals [11] and inpatient wards [21] from the staff’s perspective. It has been psychometrically evaluated in different societies, and its validity and reliability have been confirmed [13, 19]. Immense literature in this field indicates the tool’s validity and high comprehensiveness for assessing safety in healthcare systems [8, 22].

However, the Persian version of this questionnaire and the results obtained from its psychometric evaluation in various studies in Iranian society indicate instability in the factor structure of the questionnaire. Moghri et al. (2012), confirmed the 12-dimension model among the staff working in 4 hospitals affiliated to Tehran University of Medical Sciences [23]. However, the results of the study by Arabloo et al. (2012) showed that the 12-dimension model did not appropriately fit the data among the staff of the educational hospitals [15]. In another study in 2020, although the 12-dminesion model fit the data, the distribution of the items in the dimensions (except two dimensions) was reported to be different from the original version. The results of internal consistency were reported to be unacceptable for all the dimensions of the questionnaire, except for the dimension *frequency of events reported* [18]. The results of these Iranian studies are challenging and make it difficult to use the results. In this regard, conducting another survey to ensure the psychometrics properties is necessary.

On the other hand, designing and conducting research in the field of neonatal safety in neonatal intensive care units requires a valid and reliable tool that can investigate safety culture as a concept completely dependent on the culture and the society under study [9]. Therefore, to measure it, there is a need for a native tool derived from the culture of Iranian society to be able to measure its actual amount from the staff’s perspective.

In addition, several studies have shown the need to study the psychometric properties of this tool in different parts of health care [14, 18], and emphasized the evaluation of the extent and the level of safety culture in order to improve the level of patient safety and provide optimal care [10, 24].

Since safety culture is a concept that is completely dependent on culture and society and is an important factor regarding infant safety, and considering the special conditions of NICUs and the high-risk infants admitted to them, as well as the increased prevalence of errors and infant harm in the above-mentioned wards, and as the psychometric properties of this tool in the NICUs in Iran have not been reported yet, and for the results of the studies in different wards and hospitals have shown different factor structure for the tool, this study aims to investigate the psychometric properties of the Persian version of the HSOPSC among the nurses and the physicians working in NICUs.

## Methods

### Study design

This methodological research design was used to investigate the psychometric properties of the Persian version of HSOPSC among NICUs staff. This study was carried out through a census sampling of total of physicians and nurses working in NICUs in the educational hospitals affiliated to University of Medical Sciences, in Tehran, Iran, from May to September 2019.

### Setting

This study was conducted in the most prominent educational and treatment hospitals in the Tehran metropolis. These 14 hospitals (including 17 NICUs) are considered the most equipped centers to provide intensive and specialized care for premature neonates with various life-threatening problems. Of all staff, 422 physicians and nurses’ staff were working in these NICUs.

### Participants

The population included professional staff from NICUs. The eligible individuals identified from 422 physicians and nurses working in NICUs. They include the specialists and the assistants in pediatrics and the subspecialty of neonatal medicine who were physically and mentally healthy (based on their medical records), as well as the nurses holding at least a bachelor’s degree and one year of working experience in NICUs under different employment statuses (permanent, independent contractor nursing agency, and conscription plans) with physical and mental health. Managers and supervisors were excluded from the study.

### Instruments and measures

HSOPSC is a self-administered questionnaire which examines the patient safety culture with 42 items in 12 dimensions. It also has two open-ended questions, including “No events reported”, and “Patient safety grade”. The items are rated on a five-point Likert scale: completely disagree [5] to completely agree [1]. The obtained score is calculated through finding the percentage of positive response to the questions of each dimension (agree/completely agree and always/often) and dividing it by the number of questions in the same dimension, and according to the percentage of each dimension. A few negatively worded items were coded in reverse.

This questionnaire was translated from English to Persian with the permission of the United States Agency for Healthcare Research and Quality [25] and all the methods of translation and back-translation were performed in accordance with the translation guidance documents^1^. A copy of the final English version of the scale was sent to the tool designer for confirmation. The Persian version has been used for psychometric analysis. The assessment of the psychometric properties was as follows:

#### Content validity

For qualitative content validity, 10 experts in the field of safety and health of high-risk infants in nursing (2 matrons and 3 head nurses), medicine (1 neonatologist and 2 pediatricians), and tool psychometrics (2 experts), who were selected through purposeful sampling. The items were assessed in terms of simplicity, clarity, necessity and relevance [26].

#### Face validity

The questionnaire was given to 14 selected physicians and nurses working in NICUs in a targeted manner with a diversity of work experience [27–29] to assess the qualitative face validity. The tool’s response time was estimated, too.

#### Construct validity

Confirmatory Factor Analysis (CFA) was performed [30] to investigate the factor structure of the Persian version questionnaire. This technique examines the goodness of fit between a hypothetical model and data obtained from subjects [31]. For estimating the parameters, the maximum likelihood estimation was used. In this regard, the model’s goodness of fit indices includes the Akaike Information Criterion (AIC), Non-Normed Fit Index (NNFI), Standardized Root Mean Square Residual (SRMR), Comparative Fit Index (CFI), Root Mean Square Error of Approximation (RMSEA), Goodness of Fit Index (GFI), Adjusted Goodness of Fit Index (AGFI), Normed Fit Index (NFI) and Chi-Square were examined using LISREL 8.80 software by measuring weighted least squares, considering that the items are being scored on a 5-point Likert scale.

There are diverse opinions to determine the sample size needed for CFA. Some recommended 20 subjects per factor, and others opined that in questionnaires with more than three factors, 100 to 200 samples should consider for evaluation [32, 33]. A minimum sample size of 200 individuals has been recommended, too [31]. Based on the inclusion criteria and considering the probability of sample loss, a total of 360 individuals (261 nurses and 99 physicians) were included in the study through convenience sampling method. After obtaining the necessary permission to collect data and the informed consent of the participants, the researcher provided them with a questionnaire (containing two sections of questions regarding demographic information and the tool). Data collection lasted for about four months. The data collected from 18 incompletely filled out questionnaires were removed and, finally, the analysis was performed using the data obtained from 342 participants.

#### Reliability

The Cronbach’s alpha coefficient was calculated for the whole scale and each dimension (acceptable value of α ≥ 0.60) [34]. To evaluate the stability reliability, test re-test was performed [35]. Fifteen participants (5 physicians and 10 nurses), who had been selected through purposeful sampling, were asked to complete the questionnaire on two occasions 14 days apart, and the Interclass correlation coefficient (ICC) was calculated.

### Data analysis

The demographic characteristics are described using percent and frequency (descriptive statistics). To determine the level of safety culture and its dimensions, descriptive statistical methods, independent t-test, and the analysis of variance were used in SPSS software version 21 with a significance level of 0.05. The normality of data distribution assessed with Kolmogorov-Smirnov tests.

## Results

The participants were between 23 and 54 years of age, 85.08% of them were women, and 63.45% were married. Other demographic characteristics are described in Table 1.

**Table 1:** Demographic characteristics of participants (n = 342)

Variable		Frequency (percent)
Education	Bachelor’s in Nursing Master’s in Nursing Resident Pediatrician/Neonatologist	222 (64.91) 24 (7.01) 55 (16.08) 41 (11.98)
Employment status	Tarhi^*^ Gharardad^**^ Rasmi***	34 (9.95) 216 (63.15) 92 (26.90)
Work experience in NICU	2–5 years 5–8 years More than 8 years	191 (55.85) 105 (30.70) 46 (13.45)
Shift status	Fixed Rotation	72 (21.06) 270 (78.94)

* Who undertakes ** Contract Employment *** Permanent Employment

For the qualitative face validity, minor changes were applied to the questionnaire. Based on the experts’ opinions on the qualitative content validity, amendments were made. In the CFA, the 12-dimension model with 42 items did not fit the data (Table 2).

**Table 2:** Initial and final individual CFA model fit indices

Indices	Normal Theory Weighted Least Squares Chi-Square	AIC	RMSEA	CFI	SRMR	GFI	AGFI	NFI	NNFI
CFA model									
**Twelve-factors model (42 items)**	${{\upchi }}^{2}/df$= 2.06	1852.29	0.056	0.91	0.072	0.82	0.79	0.83	0.90
**Eleven-factors model (36 items)**	${{\upchi }}^{2}/df$= 1.95	1305.57	0.053	0.93	0.069	0.85	0.82	0.87	0.92
**Acceptable values**	< 2	-	˂ 0.08	≥ 0.90	˂ 0.08	> 0.95	≥ 0.90	≥ 0.90	≥ 0.90

**Fig. 1:**
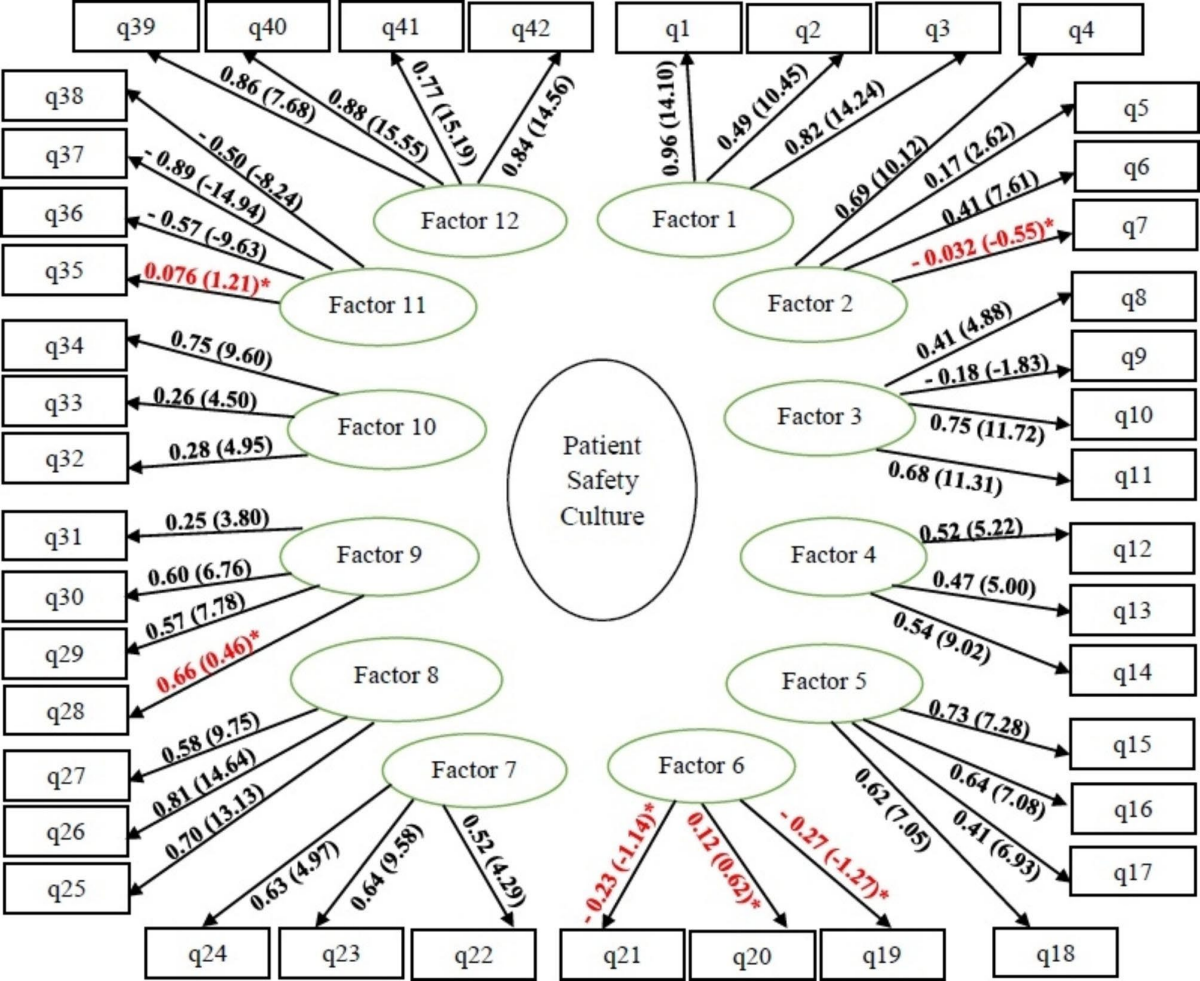
Confirmatory factor analysis of the dimensions of the Persian version of HSOPSC and its relationship with the items (the 12-factors model with 42 items)

Therefore, the items with poor T-value were removed from the model: the item of *“Our procedures and systems are good at preventing errors from happening”* was removed from the dimension *Overall perceptions of patient safety*; *“We have enough staff to handle the workload”*, from *Staffing*, *“Hospital units do not coordinate well with each other”*, from *Teamwork across units*; and all the three items, from the dimension *Communication openness* (Fig. 1).

**Fig. 2:**
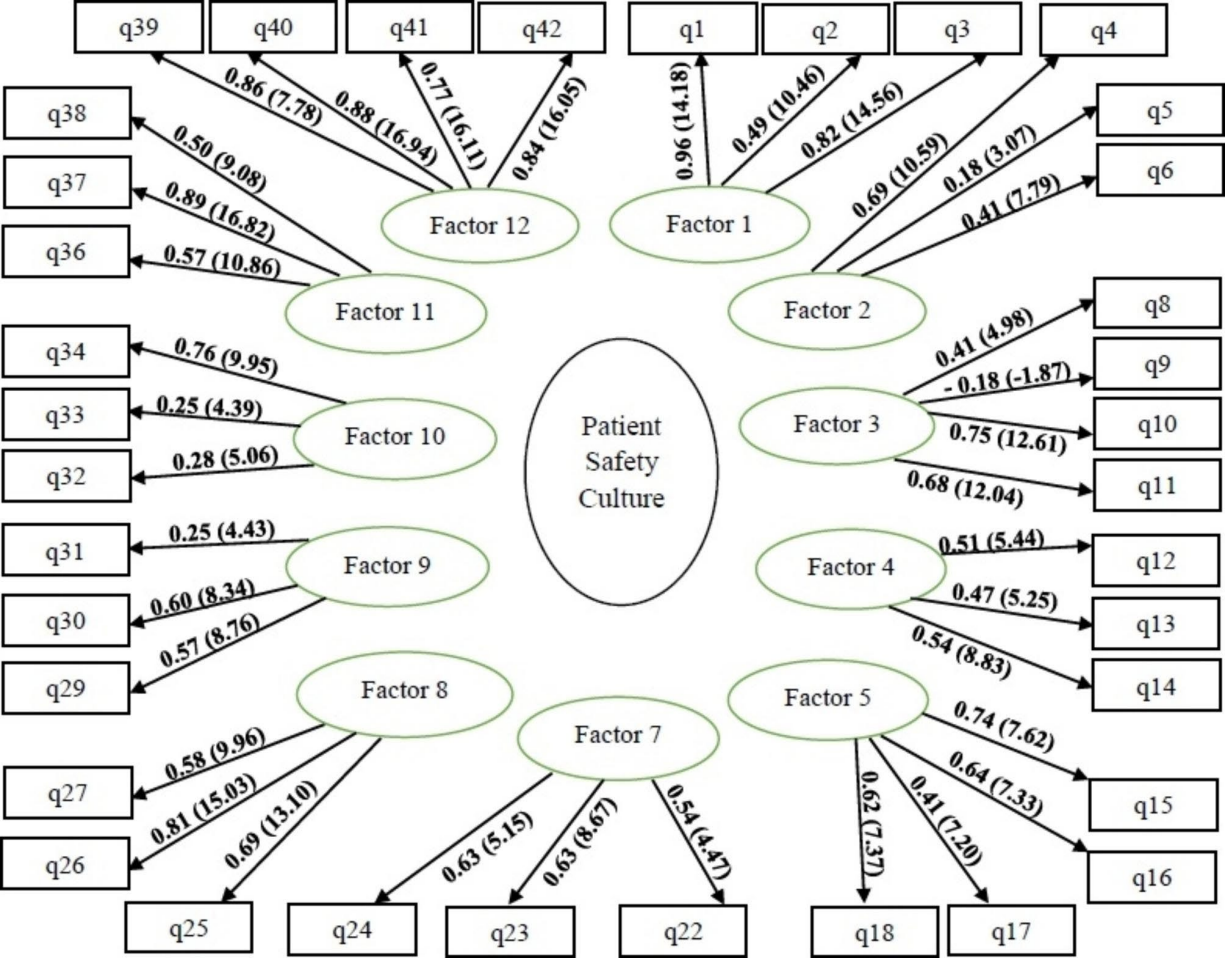
Confirmatory factor analysis of the dimensions of the Persian version of HSOPSC and its relationship with the items (the 11-factors model with 36 items)

Therefore, 6 items and one of the dimensions of the questionnaire were omitted, and CFA was performed on the obtained model. For this 11-dimensions model with 36 items, according to the T-value measured by LISREL software (Fig. 2), all the correlations between its dimensions, and items were significant and no heterogeneity was observed.

In general, according to the indices obtained in CFA (Table 2), the model and its constituent concepts are acceptable, and HSOPSC with 36 terms and 11 dimensions showed appropriate fit in the physicians and the nurses working in NICUs. The Cronbach’s alpha of the tool items was 0.79, and the ICC was 0.82 (p < 0.001) (Table 3).

**Table 3:** Average positive response rate for patient safety culture and internal consistency of the final model (11-factors, 36 items)

Dimensions	Num. Items	Percentage of positive responses patient safety culture (٪)	Mean±SD	Cronbach’s α	ICC	95% CI
Frequency of events reported	3	48.22	49.24 ± 19.61	0.80	0.79	0.72–0.86
Overall perceptions of patient safety	3	85.15	72.20 ± 21.38	0.86	0.88	0.80–0.95
Manager expectations & actions promoting patient safety	4	59.36	56.88 ± 19.65	0.81	0.90	0.83–0.97
Organizational learning	3	58.06	55.34 ± 20.75	0.63	0.85	0.77–0.92
Teamwork within units	4	79.63	64.19 ± 18.70	0.61	0.72	0.65–0.80
Feedback & communication about error	3	53.50	52.26 ± 17.97	0.74	0.79	0.72–0.86
Non-punitive response to errors	3	51.36	51.20 ± 18.13	0.84	0.73	0.66–0.81
Staffing	3	50.00	53.21 ± 20.79	0.72	0.86	0.78–0.93
Management support for patient safety	3	60.63	57.21 ± 19.57	0.77	0.83	0.75–0.90
Teamwork across units	3	64.80	58.30 ± 19.21	0.62	0.75	0.68–0.82
Handoffs & transitions	4	67.05	59.21 ± 19.83	0.73	0.78	0.70–0.85
Total	36	61.61	57.20 ± 19.59	0.79	0.82	0.75–0.89

The mean percentage of the positive responses of the nurses and the physicians working in NICUs was 61.61%, and the dimensions of *Overall perceptions of patient safety* (85.15%) and *Frequency of events reported* (48.22%) received the highest and the lowest positive responses, respectively (Table 3). During the last 12 months, 35.4% of individuals have reported at least 1 or 2 errors occurred in the ward, and 42.4% considered moderate of the level of safety culture.

## Discussion

This study aimed to investigate the factor structure of the Persian version of HSOPSC, which examined the psychometric properties (face and content validity, construct validity, internal consistency, and stability) of this questionnaire in the population of Iranian nurses and physicians working in NICUs.

Contrary to the study of Moghri et al., which confirmed the dimensions of the Persian version of HSOPSC [23], the results of the initial CFA showed that the 12-dimension model of the tool did not fit with the data. Therefore, by removing six heterogeneous items and performing CFA again, the 36-item structure fits the data in 11 dimensions. A review of numerous studies indicates instability in the factor structure of the tool in different populations [12, 17, 36]. For instance, the 12-dimension model of the original questionnaire has been approved in European and American societies [13, 37, 38]. Moreover, its other models have been reported in different societies: 11-dimension [16, 36], 10-dimension [39, 40], 9-dimension [14, 19], 8-dimension [12, 41] structures, and even a 6-dimension one [42].

The concept of safety culture depends on the underlying characteristics associated with a particular health population, care policies, and cultural differences that govern that specific society’s health system [43]. These principle differences in different countries provide a diverse structure of HSOPSC in comparison with the original version. Also, the sample size and data obtained from relatively small sample sizes in different studies can explain this lack of sameness in factor structures compared to the original version [15]. The discrepancy in the results is explained according to the various psychometric analysis methods used [18, 23]. However, what matters is that the psychometric evaluation be done in such a way that the results can be trusted. In addition, the problems raised in the translation process should be considered, too. It is not easy to find synonyms and equivalents to preserve the meaning of some words in the original language and translate them into another language [44, 45] for survey tools designed to fit the unique characteristics and contexts of specific research populations, and the target population [12]. All these components result in diversities in the factor structure and have led to the appearance of various models of this questionnaire in different societies.

The results of this study are similar to those of the research conducted in Croatia (2014) in terms of having an 11-dimension factor structure but differ in the number of items (36 items) and their arrangement in the dimensions [16]. In the present study, six items removed following the CFA results. However, in a study conducted in Slovenia [46], based on the results of the exploratory factor analysis, three items were removed due to their low factor loads: *“After we make changes to improve patient safety, we evaluate their effectiveness”, “The staff are afraid to ask questions when something does not seem right”*, *and “Shift changes are problematic for patients in this hospital”*. The remaining 39 items formed nine dimensions. In addition, there are many studies in which several questionnaire items have been removed in order to achieve better cultural adaptation and based on the factor analysis [12, 19, 41, 44].

Reviewing the psychometric studies conducted in this field, the results related to the dimension *“Communication openness”* have been challenging among the dimensions of the questionnaire. A common feature found in these studies is that in most of them, the items in this dimension merged with the dimension of “*Feedback and communication about error*” [19, 36, 39, 41, 46]. A similar result was obtained in the Iranian study conducted by Arabloo et al. (2012), and the items of these two dimensions merged into one [15]. Moreover, in the Swedish version, some of the items of these two dimensions have joined the dimension of *“Overall perceptions of patient safety”* and formed one dimension [17]. In some other studies, these two dimensions were removed completely [46]. In the present study, as in the study of Gambashidze et al. (2019) [44], the dimension of *“Communication openness”* (Factor 6) and its related items were removed due to heterogeneity and low t-statistic.

In the present study, the item “*Hospital units do not coordinate well with each other*” was removed from *Teamwork across hospital units*. Besides, the item “*Our procedures and systems are good at preventing errors from happening*” was removed from the *Overall perceptions of safety* dimension and “*We have enough staff to handle the workload*” was removed from the *Staffing* dimension. Thus, three items remained in each of these dimensions. The Romanian study achieved similar results, too. In this version, in addition to these items, all the questions in the dimensions of *“Overall perceptions of safety”* and *“Staffing”* were excluded from the questionnaire [19]. Moreover, in a recent Korean study, by removing an item from the *Staffing* dimension, the fit indices of the model were reported to be acceptable according to CFA [47]. In general, although there is no structure with similar dimensions in the conducted studies, in cultural adaptation, some joint dimensions have been obtained that correspond to the dimensions proposed in the original HSOPSC model [12, 44]. In the present study, the whole 11 dimensions and their items are similar to those of the previous studies [11, 23].

Although in almost all studies, despite the structural differences in the number of dimensions and items, the theoretical form and the content of the dimensions are preserved, in the Georgian version of the tool, a completely different structure of safety culture is seen [44]. It is the only study which introduced a 5-dimensional version of this tool. In this version, using EFA, safety culture is known as a five-dimension structure including *“Hospital-wide cooperation and support”*, *“Staff’s active role in promoting patient safety”*, *“Frequency of reported events”*, *“Teamwork within units”*, and *" “Supervisor/manager expectations and actions promoting patient safety”*. In this model, the items with negative meanings have been removed. The authors of this article infer that the participants in the study perceive and interpret the positive and negative sentences differently, and this significant point can affect other versions translated into other languages ​​as well. Confirming this conclusion, in a study, Moghri et al. have also pointed out the balance found in positive and negative words in items to facilitate the measurement of the reliability of the items in HSOPSC [48]. Different perceptions and interpretations of the negative items in the questionnaire are one of the reasons behind the diversity of individuals’ responses, which has led to the emergence of structural diversity in the tool.

In the present study, as in the initial tool development survey [10] and the research of Moghri et al. [23], Cronbach’s alpha coefficient of the tool was acceptable, which indicates the homogeneity of the items and the internal consistency of the dimensions. Regarding the dimensions of *Organizational learning-continuous improvement* and *Teamwork within/across hospital units*, Cronbach’s alpha coefficient was low, as in the Bulgarian version [38]. The dimension “*Organizational learning-continuous improvement*” is reported as unacceptable or low in several studies [14, 18, 49]. In addition, in many of the previous studies, internal consistency has been lower than in the original questionnaire in most areas [12, 44], which could be due to the differences in the number of items in these dimensions. Among these, can mention the studies conducted in Japan and Taiwan, in which Cronbach’s alpha coefficients were 0.47–0.88 and 0.26–2.83, respectively [50]. In other psychometric evaluations performed in Iran, the alpha coefficients obtained for the dimensions of the questionnaire were unacceptable and questionable [15, 18]. Regarding the test-retest results, this questionnaire has proper stability.

Although the mean percentage of positive responses from the participants in this study was 61.61%, most individuals (42.4%) considered the safety culture status moderate, which was lower than expected. It seems that the lack of manpower and the increase in the staff’s level of expectation regarding paying attention to the issue of safety in the ward has led to this contradiction; the standard number of staff employed in NICUs is an influential issue in providing safe care, and in case of its shortage, the staff will show more sensitivity about care provision for high-risk neonates. This contradiction can also be the result of individuals’ answering hastily. Because in self-report surveys with multiple questions, participants are likely to respond impatiently [51]. It is suggested that more studies be conducted to examine this issue more deeply. The necessity to design and implement more extensive quantitative and qualitative studies in this field, considering its importance, has been emphasized in recent research, too [12, 19], since setting the issue of safety as a priority is evidence of a safety culture that can overshadow all the staff’s activities, as a vital component of care provider organizations.

The lowest mean percentage of positive responses belonged to the dimension *“Frequency of events reported”*. Correcting mistakes before harming a patient requires encouraging the staff to report errors and review them accurately, while fear of the consequences of error reporting and being reproached or reprimanded by the management are the most significant obstacles to error reporting in neonatal intensive care units [52]. Therefore, trying to create a culture free of reprimand and punishment for reporting errors has been agreed upon by the questionnaire designers and the experts in this field [10, 12, 44]. Therefore, managers must make more efforts to facilitate this influential issue.

The mean score related to the “staff” is among the dimension that has been reported to possess low means in various studies [18, 53, 54]. For instance, in a recent Korean study, this dimension had the lowest positive responses from the staff perspective (13%) compared to the American version (56%) [47]. The dimension *“Staffing”* obtained a low percentage in the present study. Since management plays a vital role in creating a safety culture, it must manufacture the perspective among the staff, through taking specific measures, that safety-related issues are a high priority. One of these measures is to take a closer look at the issues related to the staff and discuss and exchange views with them regarding patient safety issues to better remove the existing obstacles and problems.

In addition, safety culture was evaluated significantly different and higher from the perspective of the residents in comparison to other participating physicians and nurses with different levels of education, and individuals with less work experience in the NICU considered safety culture to be better. This result could be because the physicians and the nurses working in the NICU with more working experience have had more contact with patients and have more severe stress due to the lack of manpower. Therefore, they tend to report a lower quality of safety culture.

## Conclusion

Overall, the results showed that the Persian version of HSOPSC has desirable psychometric properties (validity and reliability) and will be able to accurately reflect the level of safety culture regarding the local context of health care workers in neonatal intensive care units. The results of this study can help health system managers understand the status of safety culture, identify the facilitating factors and use its dimensions to make efforts to ensure the health and the safety of high-risk infants by examining the strengths and the weaknesses and better identifying the opportunities for enhancing interventions.

### Limitations

One of the limitations of this study is the effect of environmental and personal, mental, and psychological conditions of physicians and nurses on answering the self-report questionnaire and the results obtained, an issue that has been beyond the control of the researcher.

Although the results of this study showed that the Persian version of HSOPSC, with 36 items in the form of 11-dimensions, has appropriate psychometric properties and can be used as a valid and reliable tool, the safety culture can be affected by the different cultural and caring conditions of Iran. Therefore, it is suggested to design more studies to develop a suitable tool through a mixed-method approach to assess the safety culture in NICUs.

## Data Availability

The data that support the findings of this study are available on request from the corresponding author.

## Footnotes

1 Available on the AHRQ Web site: https://www.ahrq.gov/sops/international/index.html.

## Abbreviations

HSOPSC: The Hospital Survey on Patient Safety Culture
NICUs: Neonatal Intensive Care Units
CFA: Confirmatory Factor Analysis
ICC: Interclass Correlation Coefficient
AIC: The Akaike Information Criterion
NNFI: Non-Normed Fit Index
SRMR: Standardized Root Mean Square Residual
CFI: Comparative Fit Index
RMSEA: Root Mean Square Error of Approximation
GFI: Goodness of Fit Index
AGFI: Adjusted Goodness of Fit Index
NFI: Normed Fit Index
